# Effect of guideline-concordant, appropriate, and effective antimicrobial treatment on patient outcome in multidrug-resistant (MDR) infections

**DOI:** 10.1128/spectrum.00291-25

**Published:** 2025-09-08

**Authors:** Fuad A. Sindi, Abdullah Boubsit, Eyad Alkhayat, Alhasan Daghestani, Omar Albaradie, Muhammad Anwar Khan, Nour Shamas, Sharif Hala, Abdullah Awadh

**Affiliations:** 1 King Abdullah International Medical Research Center 309817, Jeddah, Saudi Arabia; 2 College of Medicine, King Saud Bin Abdulaziz University for Health Sciences, Jeddah, Saudi Arabia; 3 Infectious Disease Clinical Pharmacist, Independent Antimicrobial Stewardship Policy Consultant, Riyadh, Saudi Arabia; bioMerieux Inc, US Medical Affairs, Denver, Colorado, USA

**Keywords:** multidrug-resistant infections, MDR, antimicrobial guidelines, antibiotic guidelines, guideline-concordance, adherence, effective treatment, appropriate treatment, outcome

## Abstract

Recently, to achieve cure, physicians have been resorting to overuse or misuse of antimicrobials to treat resistant infections, leading to the emergence of further resistant organisms. To overcome this issue, antimicrobial guidelines have been developed. Nevertheless, recently, controversy regarding the effect of adherence to antimicrobial guidelines on patient outcomes has been raised. Additionally, in multidrug-resistant (MDR) infections, rates of susceptibility to guideline-recommended agents have been reported to be low. Hence, herein, we aim to assess the impact of guideline-concordant, appropriate, and effective antimicrobial regimens on patient outcomes in MDR infections at the Ministry of National Guard Health Affairs (MNGHA), Jeddah, Saudi Arabia. This retrospective cross-sectional study was conducted at MNGHA, a 500-bed tertiary care hospital in Jeddah, Saudi Arabia. Saved microbiology lab samples that grew ESKAPE pathogens (*Enterococcus* spp., *Staphylococcus aureus, Klebsiella pneumoniae, Acinetobacter baumannii, Pseudomonas aeruginosa*, and *Enterobacter* spp.) between October 2020 to September 2022 were collected and evaluated consecutively for MDR. Charts of all identified MDR isolates who had received empiric therapy were evaluated for concordance with hospital antimicrobial guidelines. Any deviation was evaluated for appropriateness by two independent researchers and a third expert. Patients under the age of 18 years were excluded, as the MNGHA hospital antimicrobial guidelines only included adults. Empiric treatment was deemed appropriate if (i) it was guideline-concordant or (ii) the deviation was approved by a third expert. The expert relied on the patient’s clinical status by examining their disease severity, lab values, and physician’s notes to adjudicate the appropriateness of the empiric treatment. If the administered antibiotic showed *in vitro* susceptibility, therapy was considered effective. The assessed outcomes included infection-associated death and clinical or microbiological cure. Over 2 years, a convenient sampling technique was applied to collect 569 samples. Out of these samples, a total of 405 samples with ESKAPE organisms were identified and evaluated, 121 of which were MDR. Four samples did not receive empirical treatment and so were excluded. A total of 117 samples were included in the analysis. The mean age in this study was 62.39 ± 18.17 years. Around three-fourths (77.8%, 91/117) of patients suffered from bacteremia, with the most common source of infection being complicated urinary tract infection. Guideline-concordant (68/117), appropriate (80/117), and effective (47/116) treatment demonstrated no statistically significant association with patient outcomes (*P* = 0.240, *P* = 0.526, and *P* = 0.747, respectively). Patients who required dose adjustment for initial empiric therapy received guideline-discordant empiric treatment more often (*P* = 0.003). Dose adjustments were judged based on hospital renal dosing guidelines and based on the patients’ creatinine clearance. Basing the empirical treatment on previous susceptibility results elevates the odds of administering effective empirical treatment (OR: 2.44, CI: 1.06–5.60, *P* = 0.033). Interestingly, guideline-concordant treatment was associated with receiving ineffective empiric treatment (OR: 0.40, CI: 0.19–0.87, *P* = 0.019). Guideline-concordant, appropriate, or effective empiric antimicrobial treatment was not associated with better outcomes for patients with MDR ESKAPE organisms. Moreover, the administration of guideline-concordant treatment showed increased odds of giving ineffective treatment. On the other hand, considering previous susceptibility results in the decision of empiric regimens was associated with a higher chance of effective antibiotic administration. This study emphasizes the importance of identifying local MDR risk factors, utilizing previous culture reports, risk stratification, and continuous evaluation of outcomes to develop hospital-specific antimicrobial guidelines to manage the challenges of providing optimal empiric antimicrobials for patients with MDR infections.

**IMPORTANCE:**

This study evaluates the impact of guideline-concordant, appropriate, and effective antimicrobial treatment on patient outcome in MDR ESKAPE infections. Our study introduces a classification differentiating between guideline-concordant and clinically appropriate treatment to provide practical insight into the complexity of decision-making in MDR infections. Additionally, this study emphasizes the importance of identifying MDR risk factors, utilizing previous culture reports, risk stratification, and continuous evaluation of outcomes to develop hospital-specific guidelines to optimize empiric antimicrobials for MDR infections.

## INTRODUCTION

Through various biochemical mechanisms, several pathogens, such as “ESKAPE” pathogens, possess the ability to resist antimicrobial therapy. ESKAPE is an acronym for a group of bacteria, including gram-negative bacteria (*Pseudomonas aeruginosa*, *Acinetobacter baumannii*, *Klebsiella pneumoniae*, and *Enterobacter* species) and gram-positive bacteria (*Staphylococcus aureus* and *Enterococcus* species). Two major concerning issues relating to ESKAPE pathogens should be highlighted. First, they are the most common contributors to nosocomial infections, and they often have the ability to resist the most commonly used antibiotics and can be biofilm producers, making them even harder to treat (1, 2). Additionally, ESKAPE pathogens are universally linked to devastating outcomes, such as prolonged hospital stay and increased morbidity and mortality, especially in intensive care units (ICUs) and immunocompromised patients (3). Antimicrobial resistance can be innate or acquired. An example of innate resistance is vancomycin resistance in *Escherichia coli*. On the other hand, acquired resistance genes can be a consequence of different types of mutations (4). These mutations are linked to the widespread use of antimicrobial agents, leading to the accelerated development of multidrug-resistant (MDR) bacteria (5). The impact of MDR bacteria is concerning, as they lead to increased hospitalization rates, length of stay, and mortality rates. The spread of ESKAPE pathogens and fear of the resistance they harbor promote inappropriate administration of broad-spectrum regimens and, consequently, lead to infection outbreaks and adverse economic effects (6).

Antimicrobial misuse among physicians is a major modifiable risk factor for the emergence of MDR organisms (7). Treating a positive culture in the absence of clinical signs and symptoms manifesting an infection, refusing to narrow the antimicrobial therapy when a causative organism is identified, and overusing a single agent or class of antibiotics in a single institute or healthcare system are some examples of antimicrobial misuse (8).

In order to control the emergence of MDR bacteria and optimize antimicrobial use, antimicrobial stewardship programs (ASPs) have been established. ASPs encompass a variety of coordinated interventions, including antimicrobial policies and behavioral change interventions (9, 10). Generally, ASP consists of several strategies and elements, such as antimicrobial guidelines. These guidelines are ideally based on the finest evidence collected from a thorough literature review and consultations with experts. Antimicrobial guidelines are formed to direct the physician to prescribe the appropriate empirical antimicrobial regimen (11). Due to epidemiological differences in antibiotic susceptibility, it is important for hospitals to develop their own antimicrobial treatment guidelines, particularly for hospital-acquired infections, basing them largely on national guidelines and adapting them to local susceptibility data (12, 13).

The importance of adherence to the antimicrobial guidelines lies in optimizing the treatment results and reducing the risk of adverse events, especially in patients with severe infections. It has been established that patients with sepsis have reduced mortality rates when proper empirical antibiotic treatment is started early (14). Controversy regarding guideline adherence and its effect on patient outcomes has been raised. On one hand, a body of evidence reported that guideline-concordant empirical therapy was associated with improved survival and 30 day mortality, while non-adherent empirical therapy demonstrated no added clinical benefit (13, 15, 16). On the other hand, opposing evidence suggests that guideline-concordant empiric therapy was not associated with any improvement in mortality, while as per hospital length of stay, some suggest reduction (17) and others report no association (18). Nevertheless, these differences might be a result of the different methodological approaches and study designs, guidelines, epidemiology, and patient population.

In complicated cases and severe infections, such as MDR infections, guidelines may not be as effective as intended. Therefore, an expert opinion that depends on individual patient scenarios and relevant investigations is considered, along with guidelines’ recommendations, to determine the appropriateness of physicians’ prescriptions (19, 20).

Notwithstanding physicians following guidelines’ recommendations or administering appropriate empirical treatment, the probability of a patient receiving an effective treatment that shows susceptibility to the causative organism is relatively low in MDR infections (2, 21).

As highlighted previously, (i) the effect of guideline concordance on patients’ outcomes was controversial; (ii) cases of MDR infections demonstrate higher complexity and, thus, may hinder guidelines’ efficacy, hence requiring an expert opinion to assess the appropriateness of empiric treatment; and (iii) low rates of susceptibility in MDR infections despite guideline-concordant therapy were observed. Therefore, this study evaluated guideline concordance, appropriateness, and effectiveness of antimicrobial therapies and their impact on patient outcomes in MDR infections in a tertiary center.

## MATERIAL AND METHODS

### Study design and setting

This retrospective cross-sectional study was conducted in the Ministry of National Guard Health Affairs (MNGHA), a tertiary hospital in Jeddah, Saudi Arabia. It is a government-funded hospital with 500-bed capacity that provides healthcare to 2.5 million national guard affiliates and their families.

### Sample collection

The study included samples of MDR bacteria collected from patients receiving healthcare at MNGHA. The specimens were sent to the in-house diagnostic microbiology lab for culturing and antimicrobial susceptibility testing routinely. Patients with MDR infections treated at MNGHA who received empiric antimicrobial therapy were included regardless of the outcome, e.g., death within a short period of time following the administration of empiric therapy. Patients suffering from MDR infections who did not receive empiric therapy or whose samples were contaminated were excluded. Infants and children under the age of 18 were excluded as the current antimicrobial guidelines do not apply to this age group.

Regarding the sample size calculation, assuming a confidence level of 95%, a margin of error of 5%, and a population of more than 20,000, the sample size was calculated using Raosoft, Inc. to be 377. The sample size did not reach this limit due to time limitations. The convenience sampling technique was applied to collect samples.

MNGHA lab used the VITEK 2 system for susceptibility testing to assess the sensitivity of the organism to certain types of antibiotics. If a current episode of infection demonstrates a syndrome that is similar to a previous infection, the susceptibility results of the previous infection were utilized to guide the decision on the choice of empiric therapy for the current infection. Molecular genetic testing was conducted using the Carba-R assay to evaluate the presence of the five carbapenemase genes: *Klebsiella pneumoniae* carbapenemase (KPC), New Delhi metallo-beta-lactamase-1 (NDM), Verona Integron-encoded Metallo-β-lactamase (VIM), Imipenemase (IMP), and oxacillinase (OXA)-48-like in *E. coli, K. pneumoniae,* and *Enterobacter* spp. only when ordered by physicians or microbiologists. These procedures are performed routinely for all samples collected from patients receiving healthcare at MNGHA.

### Definitions

According to the Centers for Disease Control (CDC) and European Centers for Disease Control (ECDC), an MDR organism is labeled as such if it has the ability to resist at least one agent in three or more antimicrobial categories (22, 23). Empirical therapy was labeled as such if it was administered prior to the report of susceptibility results. Thereafter, i.e., following the report of susceptibility results, the antibiotic regimen was labeled therapeutic. If the patient was on multiple antibiotics, doctor notes, consultation reports, and previous orders were taken into consideration to determine the empiric antibiotic regimen. In case of inconsistencies, physician’s notes, followed by infectious disease consultations, were prioritized to determine the type of infection and the administered empirical antibiotic. The antimicrobial treatment was considered guideline-concordant if the administered antibiotics corresponded to those mentioned in the pre-existing MNGHA guidelines or based on previous culture susceptibility. MNGHA Antimicrobial Guidelines were prepared based on thorough review of the literature, international guidelines, local antibiograms, MNGHA hospital formulary, and the deliberations of the members of the Corporate Antimicrobial Committee Members, which collaborates with the local ASP, with consultation of other experts outside of the committee as deemed necessary. These guidelines aimed to optimize antimicrobial use and improve management of patients with infectious diseases at the healthcare facilities of the MNGHA. Doses and frequencies were considered adherent to guideline recommendations if they were calculated and adjusted based on the patient’s needs. In case of renal impairment, the dose and frequency must be adjusted per hospital renal dosing guidelines based on the patient’s creatinine clearance. A list of MNGHA antimicrobial guidelines used for concordance evaluation with an overview of the content is added under the Supplemental material. The treatment duration was calculated in days until the physician changed the indication, or the antibiotic was discontinued for at least 24 hours. Duration was not assessed in patients who were discharged early against medical advice. Moreover, calculating the duration was not applicable in patients with febrile neutropenia or abdominal infection who died before resolution, since it requires being afebrile and non-neutropenic for 72 hours in febrile neutropenia and source control in abdominal infection. Furthermore, since the duration for bacteremia infections was not explicitly mentioned in the guidelines, a duration of 14 ± 1 days was considered optimal and aligned with the expert opinion.

If the duration was more than 15 days in bacteremia infections, the duration was calculated from the first negative culture. If it was still more than 15 days, it was considered inappropriate and not aligned with the expert opinion.

An expert opinion was needed if the antibiotics that were administered empirically and therapeutically were not aligned with the guidelines’ recommendations. An experienced infectious diseases and antimicrobial stewardship clinical pharmacist provided the expert opinion to determine whether the treatment appropriately deviated from the guidelines’ recommendations. The expert relied on the patient’s clinical status by examining their disease severity, lab values, and physician’s notes to adjudicate the appropriateness of the empiric treatment. Appropriate treatment was defined as guideline-concordant empirically administered antibiotics or guideline-discordant empirically administered antibiotics that were acceptable based on expert opinion, whereas inappropriate treatment is defined as guideline-discordant empirically administered antibiotics that were unacceptable based on expert opinion. Effective treatment is the antibiotic given, empirically and/or therapeutically, to the patient and showed *in vitro* susceptibility.

The assessed outcomes included infection-associated death and clinical or microbiological cure. Patients were considered cured in the presence of one of the following criteria: a culture from the same infection site was negative for the causative organism after a minimum of 3 days, the physician’s notes indicated improvement in the patient’s health status, or the patient was discharged from the hospital with the infectious disease department’s approval. Death was considered infection-associated if the patient expired within 48 hours after detecting the infection regardless of a negative culture or if a positive culture with the causative pathogen persisted until death.

### Data collection

Data were collected using the hospital’s electronic medical record with an integrated computerized provider order entry system: BestCare 2.0. The indication for empirical treatment was identified based on physicians’ notes. In case of insufficient data due to missing diagnosis or documentation, previous cultures and progress notes were reviewed to reach a diagnosis.

The collected data were organized via an electronic data-collection sheet. Data included patients’ history and demographics such as age and gender, date of admission, immune status—patients were categorized as immunocompromised if they had metastasis, recent hemotherapy, acute or chronic kidney injury, and end-stage renal disease, were on renal replacement therapy, or had acquired immunodeficiency syndrome—and kidney status. Data also included the departments in which the patients were when infections occurred. Those were classified into medicine, surgery, ICU, emergency department (ED), and outpatient. It also included the reference number of the collected sample along with the isolated organism, co-isolated organisms, and the sample collection site and date. Indications for empirical treatment were added to the data sheet, which included bacteremia, abdominal infection, febrile neutropenia, ventilator-associated pneumonia (VAP), hospital-acquired pneumonia (HAP), community-acquired pneumonia (CAP), complicated urinary tract infections, wound infections, and other. Bacteremia, specifically, was further subclassified into bacteremia of unknown origin or secondary bacteremia with the source. The date of the sensitivity testing report and the susceptibility results were also added to the data-collection sheet along with any molecular genetic results that might compromise the efficacy of some antibiotics, such as OXA-48, NDM-1, and KPC genes. Next, the empirical treatment administered to the patient was included. This includes the dose, route, frequency, and duration.

### Statistical analysis

Descriptive analysis was used to present all baseline characteristics. For categorical variables such as gender, infection ward, type of kidney impairment, immunocompromised status, and indication for empirical treatment, descriptive statistics were summarized using frequencies and percentages. Chi-squared test/Fisher’s exact tests were used for comparing the categorical variables as appropriate, whereas independent *t*-test was used to compare continuous data. A multivariate logistic regression was conducted to assess the predictors for guidelines concordance among the patients. The dependent variable was guidelines concordance, in which Yes was coded as 1 for event. The overall model was tested for fitness via the Hosmer-Lemeshow test and Nagelkerke R-squared. Bar graphs were used to represent the categorical data. Data were entered into MS Excel while Statistical Package for Social Sciences version 20.0 was used for data analysis. A *P*-value less than 0.05 was considered statistically significant.

## RESULTS

### Patient demographics and sample characteristics

**Fig 1:**
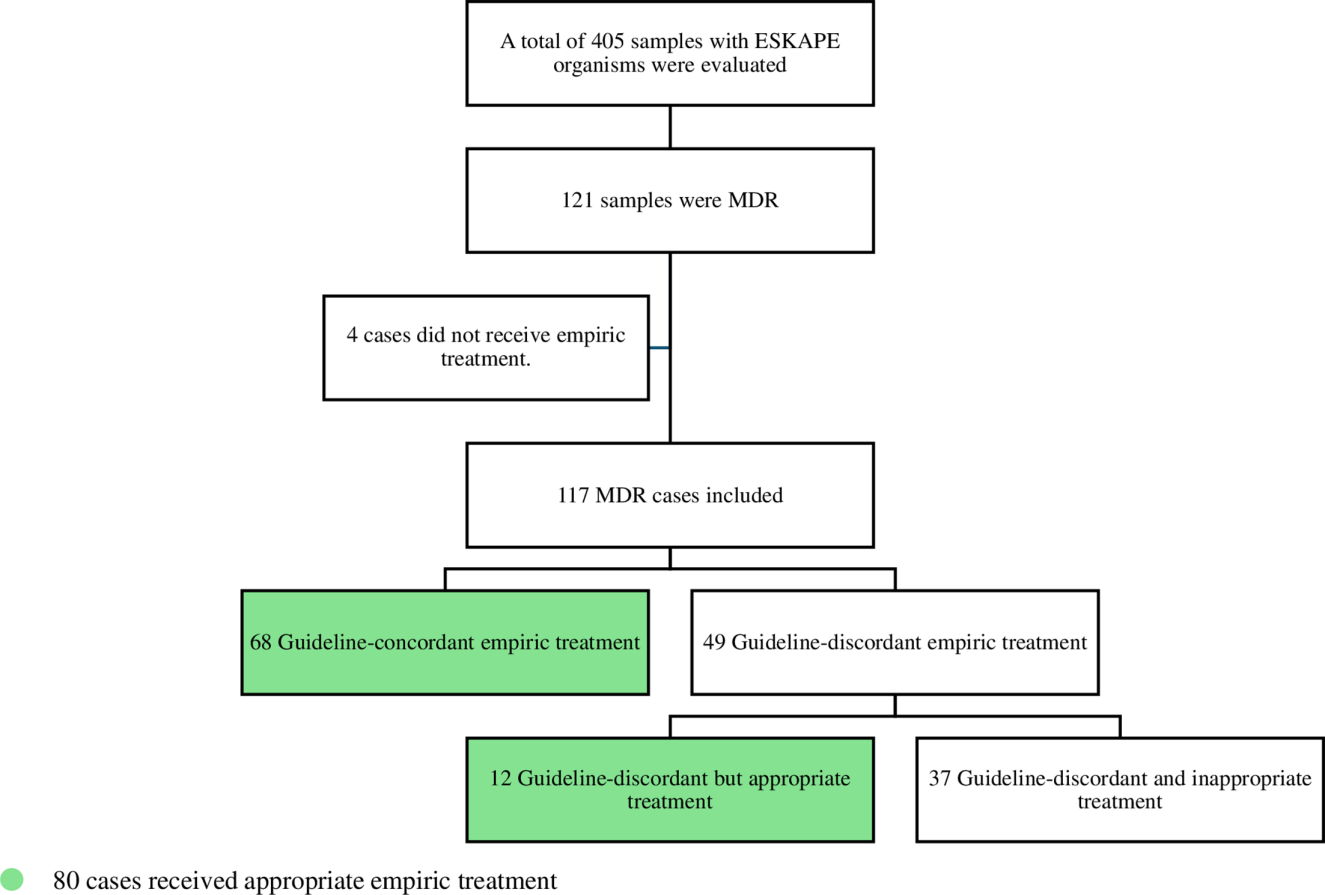
Flow diagram of sample selection and categorization.

Over 2 years, we have applied a convenience sampling technique to collect 569 samples. Out of these samples, a total of 405 ESKAPE organisms were selected from samples preserved by the microbiology laboratory between October 2020 and September 2022. Among these 405 samples, 121 were identified as MDR. One hundred seventeen samples were included in the empirical treatment analysis as the remaining four samples did not receive empirical treatment (Fig. 1). The mean age of included patients was 62.39 ± 18.17 years. The alignment between empirical antibiotic treatment and guidelines was equal in each of the medical and ICU wards (61.7%, 29/47). Most patients had renal impairment (65.8%, 77/117), with acute kidney injury (AKI) being the most common (59.7%, 46/77). Ninety-one (77.8%, 91/117) cases had bacteremia as an indication for empirical treatment followed by febrile neutropenia (8.5%, 10/117). The most common cause of bacteremia was found to be complicated urinary tract infections (20.9%, 19/91), HAP (19.8%, 18/91), and abdominal infections (16.5%, 15/91). *Klebsiella pneumoniae* was the most commonly detected organism (47.9%, 56/117), followed by *Escherichia coli* (23.9%, 28/117) (Table 1).

**TABLE 1:** The impact of patient characteristics on guideline adherence^*a*^

	Alignment between empirical treatment and guidelines					
		Guideline-concordant		Guideline-discordant		*P*
	Total *n* = 117	*n*	Mean ± SD (%)	*n*	Mean ± SD (%)	
Age	117 (62.39 ± 18.17)	72	61.028 ± 17.92	45	64.244 ± 19.168	0.360^*d*^
Gender						
Male	65	42	(64.6)	23	(35.4)	0.111^*b*^
Female	52	26	(50.0)	26	(50.0)	
Infection ward						
Intensive care unit	47	29	(61.7)	18	(38.3)	
Medicine	47	29	(61.7)	18	(38.3)	0.447^*b*^
Emergency department	17	7	(41.2)	10	(58.8)	
Surgery	6	3	(50)	3	(50)	
Kidney impairment						
Yes	77	42	(54.5)	35	(45.5)	0.277^*b*^
Type of impairment						
AKI	46	25	(54.3)	21	(45.7)	
AKI on top of CKD	15	10	(66.7)	5	(33.3)	0.704^*c*^
ESRD	11	5	(45.5)	6	(54.5)	
CKD	4	2	(50)	2	(50)	
Post-kidney transplant	1	0	(0)	1	(100)	
Renal replacement therapy						
Yes	24	13	(54.2)	11	(45.8)	0.660^*b*^
Required dose adjustment						
Yes	62	28	(45.2)	34	(54.8)	0.003^*b*^
Immunocompromised						
Yes	81	47	(58.0)	34	(42.0)	0.975^*b*^
Organism						
*K. pneumoniae*	56	33	(58.9)	23	(41.1)	
*E. coli*	28	13	(46.4)	15	(53.6)	
*A. baumannii*	12	7	(58.3)	5	(41.7)	0.497^*b*^
*P. aeruginosa*	12	8	(66.7)	4	(33.3)	
*Enterococcus* spp*.*	9	7	(77.8)	2	(22.2)	
Indication for empirical treatment						
Bacteremia	91	51	(56)	40	(44)	
Febrile neutropenia	10	5	(50)	5	(50)	
Abdominal infection	9	8	(88.9)	1	(11.1)	
HAP	2	0	(0)	2	(100)	0.107^*c*^
Catheter tip contamination	1	1	(100)	0	(0)	
Complicated urinary tract infection	1	0	(0)	1	(100)	
Invasive wound infection	1	1	(100)	0	(0)	
Non-invasive wound infection	1	1	(100)	0	(0)	
VAP	1	1	(100)	0	(0)	
Primary cause of bacteremia (*n* = 91)						
Complicated urinary tract infection	19	6	(31.6)	13	(68.4)	0.004^*c*^
HAP	18	7	(38.9)	11	(61.1)	
Abdominal infection	15	13	(86.7)	2	(13.3)	
VAP	11	9	(81.8)	2	(18.2)	
Line infection	11	6	(54.5)	5	(45.5)	
Invasive wound	7	6	(85.7)	1	(14.3)	
CAP	5	3	(60)	2	(40)	
Unknown origin	4	1	(25)	3	(75)	
Aspiration pneumonia	1	0	(0)	1	(100)	

^*a*^ CKD, chronic kidney disease; ESRD, end-stage renal disease.

^*b*^ Chi-squared test.

^*c*^ Fisher’s exact test.

^*d*^ Independent *t*-test.

### The relationship between antimicrobial treatment and patient outcome

#### Guideline-concordant treatment

**Fig 2:**
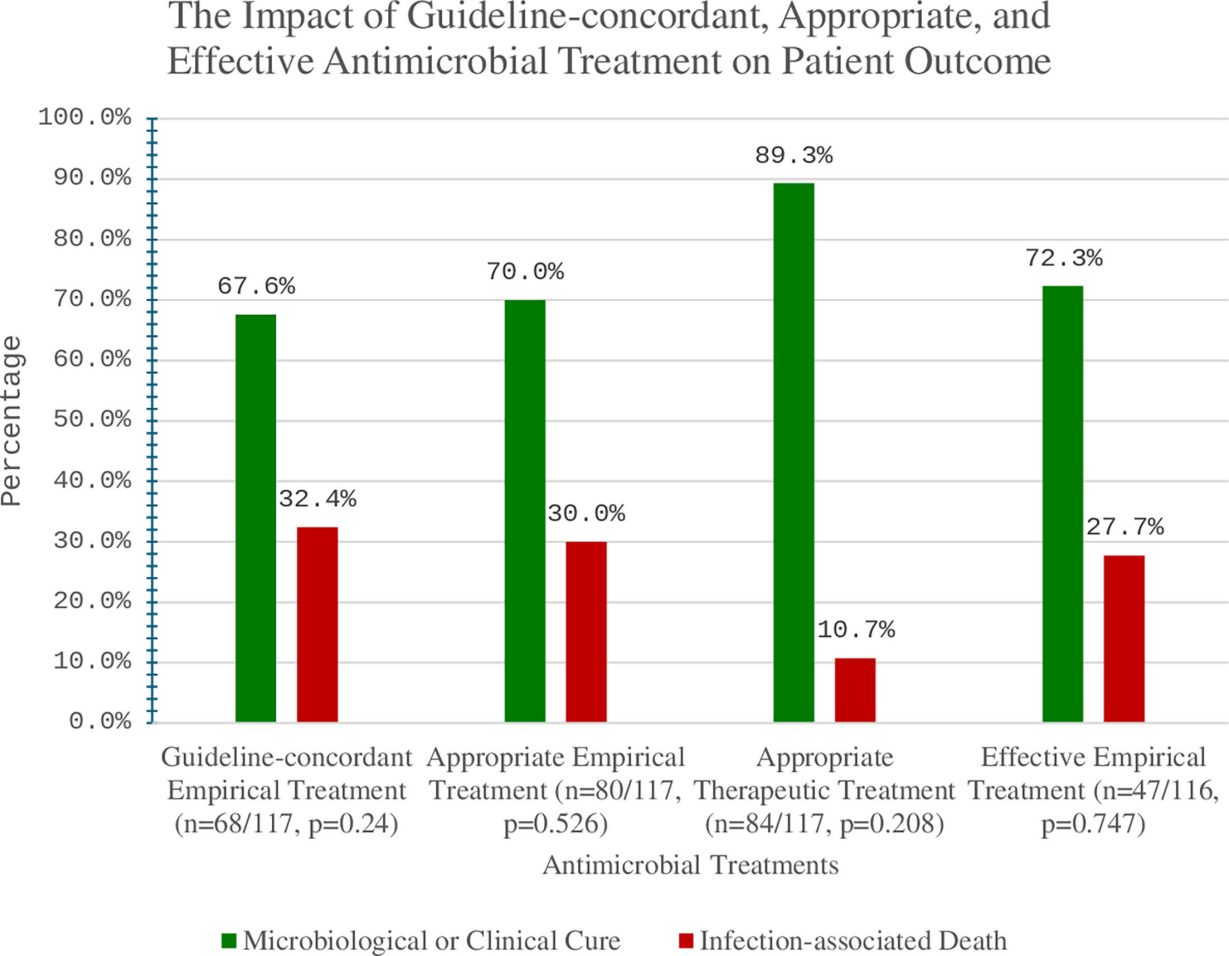
The impact of guideline-concordant, appropriate, and effective antimicrobial treatment on patient outcome.

As portrayed in Fig. 2, out of the 68 patients who were treated empirically according to the guideline’s recommendations, only 46 (67.6%, 46/68) patients had a microbiological or clinical cure. On the other hand, 22 patients (66.7%, 22/33) out of 33 patients who had an infection-associated death had received empirical treatment aligned with the guidelines (chi-squared, *P* = 0.240). Moreover, among 67 (57.3%, 67/117) patients with 90 day mortality, 36 (53.7%, 36/67) patients received guideline-concordant empirical treatment. The chi-squared test was applied to the relationship between adherence to guidelines and 90 day mortality (*P* = 0.265) (Table 2). Sixty-two (53%, 62/117) cases required dose adjustment. Cases that required dose adjustment showed lower adherence rate (45.2%, 28/62) compared to those without dose adjustment (72.7%, 40/55). A statistically significant relationship was identified between the need for dose adjustment and non-compliance with antimicrobial guidelines. The overall guideline adherence rate in bacteremia was low (56%, 51/91). The primary cause of bacteremia was found to affect the adherence to antimicrobial guidelines statistically significantly (*P* = 0.004). Of all the primary causes of bacteremia, cases of abdominal infections, invasive wound infections, and VAP reported the highest adherence rates (86.7%, 85.7%, and 81.8%, respectively). On the other hand, cases of bacteremia secondary to infections of unknown origin and complicated urinary tract infections showed the lowest adherence rates (25.0% and 31.6%, respectively) (Table 1). A multivariate logistic regression was conducted to assess the predictors for guidelines concordance among the patients. The dependent variable was guidelines concordance, in which Yes was coded as one for event. The overall model demonstrated a lack of fit as indicated by the Hosmer-Lemeshow test as 0.647. Negelkerke R-squared specifies that 70.6% of the results can be explained by the predictors. Among all the independent variables, none of them showed a statistically significant contribution to the model (Table 3).

**TABLE 2:** The relationship between guideline-concordant treatment and 90 day mortality^*a*^

			Alignment between empirical treatment and guidelines				
		Total	Yes		No		*P*
		*n* = 117	*n* = 68	%	*n* = 49	%	
90 day mortality	Yes	67	36	(53.7)	31	(46.3)	0.265
	No	50	32	(64.0)	18	(36.0)	

^*a*^ Chi-squared test.

**TABLE 3:** Multivariate logistic regression^*a*^^,^ ^*b*^

	Odds ratio	95% CI		*P*
Age	0.963	(0.90	1.03)	0.256
Gender
Male	1 (Reference)			
Female	3.609	(0.22	59.90)	0.371
Infection ward
Medicine	1 (Reference)			0.997
Surgery	486501927388608000	(0.00		0.997
Intensive care unit	1.310	(0.12	14.46)	0.825
Emergency department	24135721624700600000000000	(0.00		0.998
Type of dysfunction	
AKI	1 (Reference)			0.762
AKI on top of CKD	1.773	(0.17	18.65)	0.633
CKD	0.431	(0.01	16.30)	0.650
ESRD	0.148	(0.00	9.04)	0.362
Post-kidney transplant	0.000	(0.00		0.999
Renal replacement therapy	
Yes	1 (Reference)			
No	2.207	(0.14	34.90)	0.574
Immunocompromised	
Yes	1 (Reference)			
No	0.000	(0.00		0.997
Organism
*A. baumannii*	1 (Reference)			0.858
*E. coli*	0.000	(0.00		0.998
*Enterococcus* spp*.*	90790263	(0.00		0.999
*K. pneumoniae*	0.200	(0.01	3.12)	0.251
*P. aeruginosa*	0.265	(0.01	10.87)	0.483
Primary cause of bacteremia	
VAP	1 (Reference)			0.976
HAP	0.155	(0.01	1.98)	0.151
CAP	1354.726	(0.00		>0.99
Abdominal infection	8323133234689380000000000	(0.00		0.997
Unknown origin	0.000	(0.00		0.999
Line infection	0.224	(0.01	5.77)	0.367
Invasive wound	71835949553481900000000000	(0.00		0.998
Complicated urinary tract infection	0.202	(0.01	4.84)	0.323
Aspiration pneumonia	0.000	(0.00		>0.99
Constant	72.187			0.181

^*a*^ CKD, chronic kidney disease; ESRD, end-stage renal disease.

^*b*^ Multivariate logistic regression.

#### Appropriate treatment

**Fig 3:**
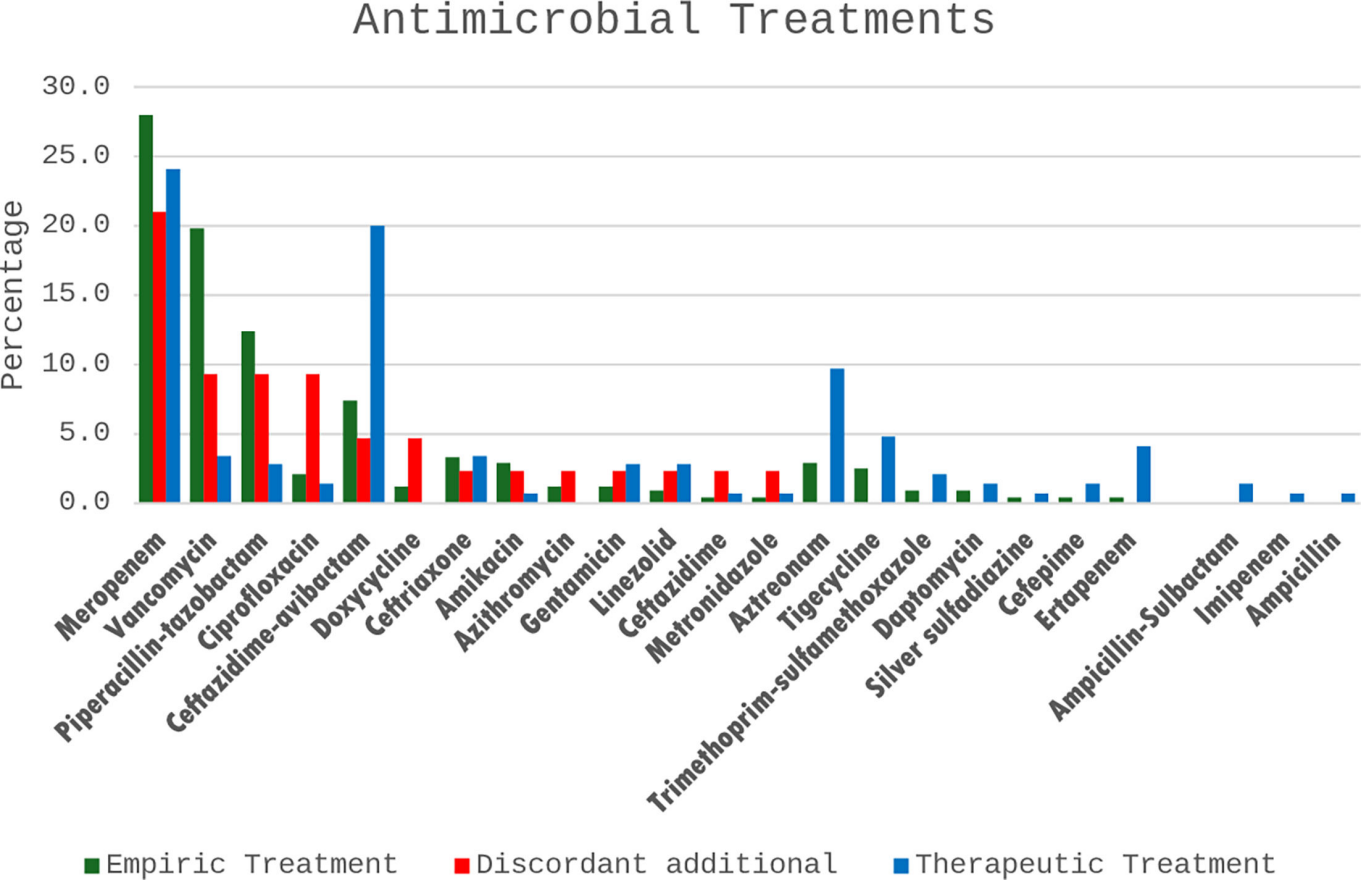
The administered empirical, discordant additional, and therapeutic antimicrobial treatments.

Appropriate empirical treatment was observed in 80 cases, with 70% (56/80) showing microbiological or clinical cure. Similarly, out of 37 cases where inappropriate treatment was used, 75.7% (28/37) demonstrated microbiological or clinical cure. The relationship between appropriate empirical treatment and patient outcome was statistically non-significant (*P* = 0.526). An appropriate therapeutic choice was reported in 84 (71.8%, 84/117) cases and resulted in (89.3%, 75/84) microbiological or clinical cure rate. The statistical relationship between appropriate therapeutic choice and patient outcome was non-significant (*P* = 0.208). Table 4 shows the alignment and deviation status. The most noted guideline-discordant action, in 34 cases (29.1%, 34/117), was the addition of a guideline-discordant antibiotic. According to the expert opinion, only 13 cases (38.2%, 13/34) deviated appropriately from the guidelines by adding a guideline-discordant antibiotic. Figure 3 shows the rate of antimicrobials administered empirically, discordantly, and therapeutically.

**TABLE 4:** Appropriate treatment and deviation

	*n*	%
Patient received therapeutic antimicrobials according to susceptibility results (*n* = 121)		
Susceptible	77	63.6
No therapeutic treatment given	25	20.7
Resistant	19	15.7
Guideline-discordant additional antibiotics (*n* = 117)		
Yes	34	29.1
Guideline-discordant missing antibiotics (*n* = 117)		
Yes	16	13.7
Frequency alignment (*n* = 117)		
Yes	107	91.5
Dose alignment (*n* = 117)		
Yes	99	84.6
Duration alignment (*n* = 117)		
The recommended duration was not mentioned in the guideline	92	78.6
Yes	15	12.8
Not applicable	9	7.7
No	1	.9
Deviated by adding antibiotics (*n* = 34)		
Acceptable deviation	13	38.2
Deviated by missing antibiotics (*n* = 16)		
Acceptable deviation	6	37.5
Deviated in frequency (*n* = 10)		
Acceptable deviation	1	10.0
Deviated in dose (*n* = 18)		
Acceptable deviation	2	11.1
Deviated in duration (*n* = 62)		
Acceptable deviation	40	64.5
Deviated in therapeutic choice (*n* = 20)		
Acceptable deviation	7	35.0

#### Effective treatment

**Fig 4:**
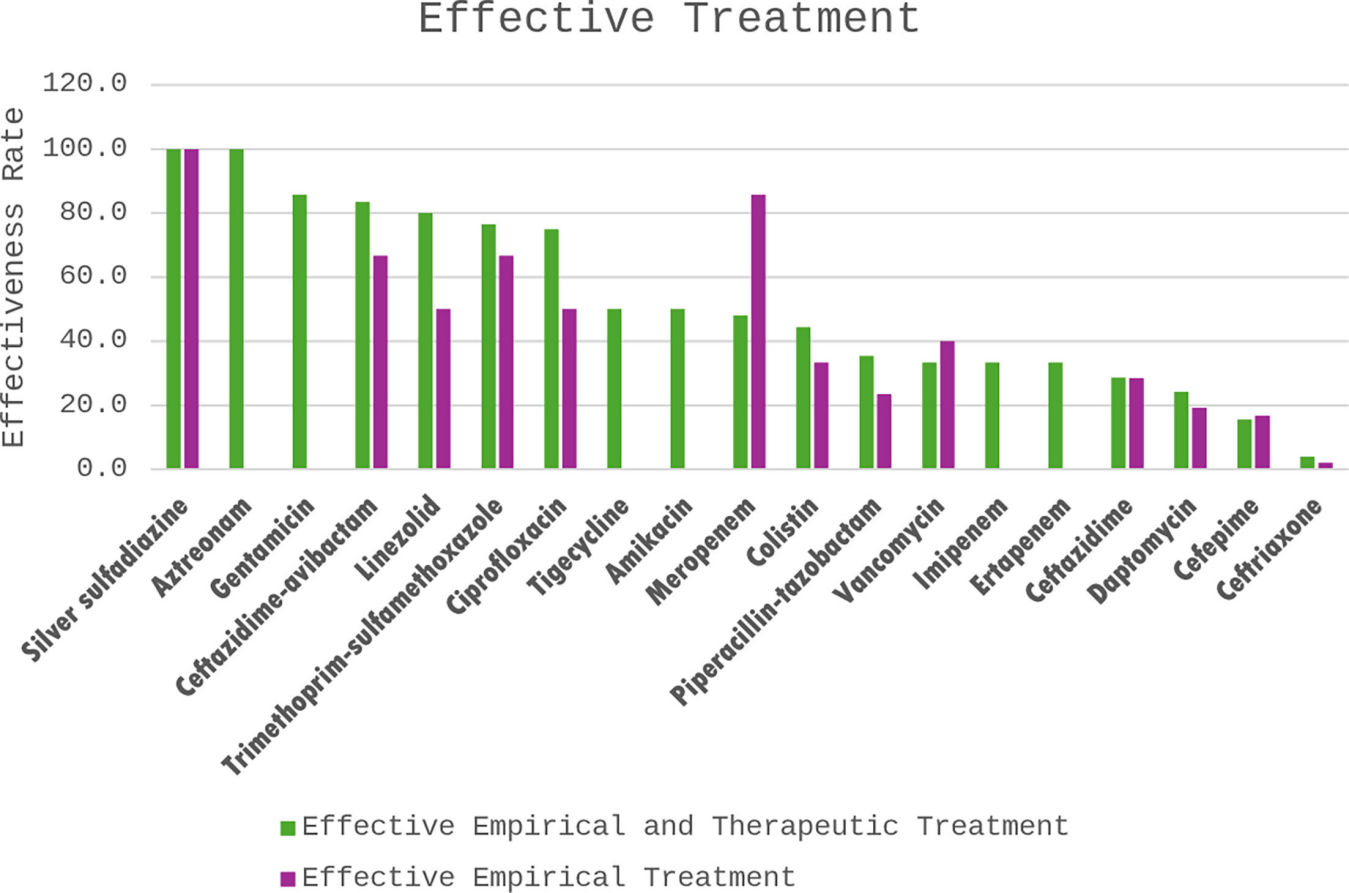
The effectiveness rates of the administered antimicrobial treatments empirically and therapeutically.

In this study, 116 cases were evaluated for effectiveness. A comparable microbiological or clinical cure rate was found in cases where either effective or ineffective empirical treatment was administered (72.3% and 69.6%, respectively). Effective empirical treatment was not associated with microbiological or clinical cure (*P* = 0.747). Table 5 shows that patients who received guideline-concordant empirical treatment resulted in a low effectiveness rate (31.3%, 21/67). Guideline-concordant treatment was more likely to lead to ineffective treatment (OR: 0.40, CI: 0.19–0.87, *P* = 0.019). Moreover, previous microbiological data were utilized in 32 cases and resulted in a 56.3% (18/32) effectiveness rate. The use of empiric treatment based on previous sensitivity results was more likely to lead to effective treatment (OR: 2.44, CI: 1.06–5.60, *P* = 0.033). Figure 4 describes the effectiveness rates of the administered antibiotics. The susceptibility report was recorded, together with its relationships with the guideline’s recommendation and the empirical treatment (see Supplemental material).

**TABLE 5:** Factors influencing the effectiveness of empirical treatment^*a*^

		Effective empirical treatment							
		Effective empirical treatment		Ineffective empirical treatment		OR	95% CI		*P*
	Total (*n* = 116)	*n*	%	*n*	%				
Treatment based on previous susceptibility reports									
Yes	32	18	(56.3)	14	(43.8)	2.44	(1.06	5.60)	0.033
Alignment between empirical treatment and guidelines									
Yes	67	21	(31.3)	46	(68.7)	0.40	(0.19	0.87)	0.019

^*a*^ Chi-squared test.

## DISCUSSION

This study concluded four main findings: (i) out of 68 (58.1%, 68/117) cases that received guideline-concordant empirical treatment, 46 had a clinical or microbiological cure. The relationship between following the guidelines in treating MDR organisms empirically and patients’ outcomes was statistically non-significant (*P* = 0.240). (ii) Appropriate empirical treatment was administered to 80 patients and resulted in 56 (70.0%, 56/80) clinical or microbiological cure, yet there was no statistically significant difference between either receiving appropriate or inappropriate empiric antimicrobial regimens on the outcome. (iii) The administered empirical treatment was effective in 47 cases with a cure rate of 72.3% (34/47) . However, the relationship between receiving effective empirical treatment and patient outcome was statistically nonsignificant. (iv) Guideline-concordant treatment was effective in 21 (31.3%, 21/67) cases and was more likely to lead to receiving ineffective treatment. On the other hand, previous susceptibility reports were used in 32 cases, resulting in a (65.3%, 18/32) effectiveness rate, and were significantly linked to receiving effective treatment.

Herein, this study offers many advantages. Unlike the previous studies that specified a single infection or hospital department, our study did not exclude any hospital department nor focus on a particular infection to have a broader assessment of the guidelines’ applicability and physicians’ prescription behavior in various MDR cases. We thoroughly evaluated the appropriateness of use based on the available guidelines as well as expert opinion, as we expected that treatment of MDR infections is complicated and deviation from guidelines may be appropriate. This study is also one of the first regionally to specifically evaluate the impact of hospital guidelines on patient outcomes in patients with MDR infections.

### Antimicrobial treatment impact on patient outcome

This study included 117 samples in the empirical treatment analysis as the remaining 4 samples did not receive empirical treatment. The assessed outcomes included infection-associated death and clinical or microbiological cure. Furthermore, this study mainly relied more on microbiological cure since it was difficult to assess the clinical improvement for a certain infection in such settings, as the majority of patients suffered from multiple comorbidities, developed different infections with a different organism, or were generally in a critical state (24).

#### Guideline-concordant treatment

A study on healthcare-associated pneumonia with 11 (11%) cases of MDR infections and 6 (14%) cases of guideline-concordant therapy concluded that guideline compliance did not improve clinical outcomes (25), which is in line with our findings. Additionally, guideline-concordant treatment was associated with increased mortality in a study that linked guideline adherence to MDR pneumonia in 303 ICU patients (26). In contrast, a study on septic patients that included 88 (17%) MDR cases, with an adherence rate of 54.8% (44/88), resulted in 35 deaths. Even though the adherence rate was higher in these cases than in the whole sample (41.7%), the mortality rate was higher in MDR cases and concluded MDR pathogens as an independent risk for mortality (27). Another study on severe CAP patients in the ED included 630 patients with 66 MDR cases. It resulted in a total of 359 (57%) guideline-concordant treatments and 30 guideline-concordant treatments in MDR cases. Guidelines adherence improved 30 day survival rates, and MDR isolates were not independent factors for mortality (28). The variation in impact of guideline adherence on patient outcomes is likely related to variabilities in suitability of guidelines to patient population epidemiology and resistance patterns. Lack of adequate empirical antibiotic coverage that may delay receiving the effective regimen along with infection severity can explain why 22 (66.7%) cases of infection-associated deaths received guideline-concordant treatment. This is expected as infectious disease treatment guidelines are not usually catered to MDR infections.

In accordance with other studies, different types of pneumonia had different rates of adherence (29). Moreover, the relationship between the primary causes of bacteremia and the alignment with empirical antimicrobial guidelines was statistically significant. The highest alignment with empirical antimicrobial guidelines was observed in VAP, abdominal, and invasive wound infections. Our guidelines recommend meropenem (broad-spectrum antibiotic) as an empirical antibiotic for the majority of abdominal infections. On the other hand, aspiration pneumonia and bacteremia of unknown origin had the lowest alignment to empirical antimicrobial guidelines. This might be due to the fact that bacteremia of unknown origin requires more time to achieve source control. In addition, when treating sepsis of unknown origin, current guidelines advise referring to specific primary infection guidelines. This might delay source control as physicians sometimes fail to detect the source upon initial efforts. This was observed several times in our study. For example, a case of bacteremia of unknown origin was started on a pneumonia regimen as it was the suspected cause of bacteremia upon presentation of the patient to the emergency department. However, the regimen was changed to a urinary tract infection regimen after the admission of the same patient in the same episode.

A large meta-analysis found that dose adjustments are done in only 50% of the cases (30). Herein, 62 (53%, 62/117) patients required dose adjustment. More than half (54.3%, 34/62) of those patients received guideline-discordant empirical treatment. Our study revealed a statistically significant association between the need for dose adjustment and misalignment with guidelines (*P* = 0.003). This indicates that physicians were less likely to adjust doses and frequencies of the administered antibiotic for patients with kidney impairment or renal replacement therapy. A reasonable explanation is that dose adjustment guidelines are not found within the same antimicrobial guidelines. Rather, they are found in another separate document, which can be accessed through the intranet of the tertiary center. Hence, it might be helpful to integrate the two guidelines in a single document that is easily accessible and integrate both guidelines into the computerized physician order entry (31). Furthermore, these findings suggest that it is crucial to disseminate awareness regarding the necessity of implementing dose adjustment guidelines.

#### Appropriate treatment

Although, in straightforward cases, antimicrobial guidelines demonstrate high efficacy, complicated cases, such as MDR infections, might render those guidelines underperforming. As the population in this study was patients infected with MDR bacteria, an expert opinion was considered in determining the appropriateness of patient treatment. Our results demonstrated no statistically significant difference between receiving appropriate or inappropriate empiric antimicrobial regimens (*P* = 0.526). This might indicate a shortcoming in the application of general hospital antimicrobial guidelines to complicated cases, such as MDR cases. Therefore, further investigation and analysis of local MDR susceptibility data is essential in developing comprehensive and highly effective guidelines.

Furthermore, overprescribing or overtreatment with broad-spectrum antibiotics comprises a concerning issue that antimicrobial stewardship programs are putting huge efforts to mitigate. This type of “defensive medicine” has contributed to the emergence of MDR (32). This study assessed the type of deviation from antimicrobial guidelines. Deviation from the guidelines via adding a guideline-discordant antibiotic was evident in 34 out of 117 cases (29.1%). Although the majority of these incidences were unacceptable per the expert opinion, labeling these findings as overtreatment or “defensive medicine” might be unjust as patients enrolled in this study present with complicated cases in terms of MDR and comorbidities. Moreover, the most guideline-discordant additionally prescribed antibiotic was colistin, followed by meropenem. The use of these “heavy hitters” might be attributed to the complexity of the included cases in this study. Colistin has notoriously gained a reputation for reduced safety despite high efficacy (33, 34). Nevertheless, according to Kasiakou et al., colistin demonstrated high levels of efficacy and safety in severe nosocomial infections (35). Similarly, Sobieszczyk et al. evaluated the safety and efficacy of colistin in combination with either imipenem or meropenem, amikacin, tobramycin, aztreonam, cefepime, sulbactam, or quinolone in gram-negative MDR respiratory infections. Interestingly, colistin combinations showed reasonable safety and efficacy (36). These findings also add to the necessity of thorough evaluation of the data of complex cases, such as those with critical illnesses, severe complications with uncontrolled comorbidities, and MDR infections, to develop a more comprehensive antimicrobial guideline.

#### Effective treatment

Several studies correlate MDR infections with receiving ineffective empirical treatment and mortality (37, 38), whereas other studies only link MDR infections with administering ineffective treatment (39, 40). In line with previous studies, our study had a high ineffectiveness rate (59.5%, 69/116). Compared to 72.3% (34/47) of our patients who received effective treatment and achieved a clinical or microbiological cure, 69.6% (48/69) of those who received ineffective treatment also achieved a clinical or microbiological cure. No significant association was found between effective empiric treatment and patient outcomes. In comparison, a study with 127 MDR infections reported that 82.6% of the administered empirical antibiotics were effective, although without reducing the overall mortality (41). The remarkable higher effectiveness rate is potentially related to the study hospital’s regulations, which did not restrict the use of broad-spectrum antibiotics for empiric use and which may have increased the likelihood for patients to receive broad-spectrum empiric antibiotics, compared to our hospital where some antibiotics were restricted and required Antimicrobial Stewardship team approval. Nonetheless, the lack of regulations and indiscriminate use of broad-spectrum antibiotics for all patients may result in worsening antimicrobial resistance rates and, eventually, may not have the desired impact on mortality.

In critically ill patients with MDR infections, the effectiveness of an administered treatment is low even when following guidelines’ recommendations or using appropriate therapies. This leads to a delay in administering effective therapy and, consequently, a poor outcome (2). A previously mentioned study (27) reported that 81% of guideline-concordant therapy was effective, and 85% of guideline-discordant therapy was effective, with no statistical significance. In contrast, despite the high adherence to guidelines’ recommendations, MDR infections were more likely to receive ineffective empirical treatment (41.8%) (42). Our study similarly reported that adherence to guidelines was linked to a high rate of ineffective treatment (68.7%, 46/67). This can be attributed to the fact that the hospital’s guidelines are not designed to treat MDR specifically but rather a syndromic approach to infections.

Instead of the unregulated use of broad-spectrum antibiotics to achieve a high effectiveness rate, another approach is to use the patient’s previous susceptibility reports. As reported in Fig. 4, empirically administered ceftazidime-avibactam had an effectiveness rate of more than 66.7% (12/18), although this antibiotic was not recommended for empirical use by our guidelines at the time of the study. This is an example of utilizing prior microbiological data to aid the early initiation of effective treatment. Such action is backed by similar studies that concluded that when following previous susceptibility results in MDR infections, the accuracy of the empiric treatment improved. On the other hand, ignoring previous microbiological results drastically decreased the accuracy rate of empiric therapy (43, 44). Similarly, herein, we report that use of previous sensitivity results to determine choice of empiric treatment is associated with increased rates of effective treatment. Therefore, this study recommends using previous microbiological data for previously infected MDR patients, critically ill patients, and highly suspected MDR infections to decide on optimal empiric antibiotic therapy.

To cover all possible MDR organisms empirically, it requires unrealistic and potentially unsafe routine administration of broad-spectrum combination regimens; therefore, the development and implementation of MDR-oriented local guidelines are essential. The results of this study suggest developing tailored hospital-specific guidelines based on locally identified MDR risk factors including previous culture results, risk stratification, and continued evaluation of outcomes to inform on the effectiveness of the guideline-recommended therapy. Additionally, early involvement of infectious disease experts, including infectious disease physicians and clinical pharmacists, is critical to optimize therapy.

Another critical factor to increase the likelihood of clinical or microbiological cure is the early administration of an effective therapeutic antibiotic, as suggested by several studies (2, 45). In this study, appropriate therapeutic treatment was observed in two-thirds of the patients. Despite these high appropriateness rates and increased likelihood of clinical or microbiological cure, the statistical relationship was non-significant (*P* = 0.208). These results contrast with another study that emphasized the faster implementation of effective therapeutic treatment as an important factor for mortality (45). Although the specific reasons for some physicians to ignore the susceptibility reports were unclear, these findings highlight the need to increase adherence to the susceptibility report and earlier initiation of effective antimicrobial therapy.

### Limitations

This study faced several limitations. First, we initially considered screening patients with risk factors for MDR infections for inclusion. However, due to the difficulty of identifying patients with MDR risk factors retrospectively from patient charts due to missing documentation and the broad definitions for MDR risk factors, we resorted to identifying patients for inclusion based on an objective parameter which is the positive culture result for ESKAPE pathogens. Additionally, only data from one tertiary center were utilized, therefore requiring further larger multicenter studies to generalize the results. Furthermore, due to the complex nature of our cases, MDR infections with multiple comorbidities, an expert opinion was considered to adjudicate empiric treatment regimens in spite of guidelines. This raises some concerns; specifically, non-differential misclassification due to high rate of non-concordance among experts (46), which may lead to bias toward the null. Also, as the study’s data rely on what is recorded in the hospital’s electronic database, the degree of accuracy depends on the information documented by the medical staff. Another limitation is the inability to assess the infection severity. Also, some confounding variables that might affect the outcomes, such as those of the Charlson comorbidity index, were not collected and therefore not adjusted for. Also, as many syndromes outside of bacteremia do not have tests of microbiological cure, no systemic evaluation on microbiologic cure was performed, leading to an elevated chance of selection bias. Clinical cure as an outcome might have been exposed to non-directional misclassification, leading to bias toward the null. Finally, the relatively small sample size did not meet sample size calculation, and patients’ heterogeneity can contribute to the non-significant *P*-values.

### Conclusion

This study evaluated the physicians’ adherence to the antimicrobial guidelines, the appropriate use of antibiotics, the effectiveness of the administered therapies, and the impact on patients’ outcomes in MDR ESKAPE infections. Our results found no statistically significant impact of guidelines-concordant, appropriate, or effective treatment on patient outcome. Furthermore, administering empirical treatment based on guideline recommendations was more likely to lead to ineffective treatment. On the other hand, the reliance on the patient’s previous sensitivity reports was more likely to lead to effective treatment. This study highlights the difficulties faced in ensuring optimal empiric therapy in patients with MDR infections. It also emphasizes the importance of developing tailored hospital-specific guidelines based on locally identified MDR risk factors including previous culture results, risk stratification, and continued evaluation of outcomes to inform on the effectiveness of the guideline-recommended therapy. Additionally, further investigation is necessary to assess the reasons behind guideline non-compliance. This will aid in addressing those factors and improving patient outcomes.

### Key messages

- Guideline-concordant, appropriate, or effective empiric antimicrobial treatment was not associated with better outcomes for patients with MDR ESKAPE organisms.
- The administration of guideline-concordant treatment showed increased odds of giving ineffective treatment.
- The reliance on the patient’s previous sensitivity reports was more likely to lead to effective treatment.
- The relationship between the need for dose adjustment and non-compliance with antimicrobial guidelines was statistically significant.

## SUPPLEMENTAL MATERIAL

Supplemental material spectrum.00291-25-s0001.docx Additional data on the sensitivity of antimicrobial agents and micro-organisms and a brief description of all guidelines used in the conduction of this study.

ASM does not own the copyrights to Supplemental Material that may be linked to, or accessed through, an article. The authors have granted ASM a non-exclusive, world-wide license to publish the Supplemental Material files. Please contact the corresponding author directly for reuse.
