# Supplementary material for: Effect of guideline-concordant, appropriate, and effective antimicrobial treatment on patient outcome in multidrug-resistant (MDR) infections
Source: Microbiol Spectr. 2025 Sep 8;13(10):e00291-25. doi: 10.1128/spectrum.00291-25 (PMC12502525; doi:10.1128/spectrum.00291-25)
Supplement: Supplemental material — Additional data on the sensitivity of antimicrobial agents and micro-organisms and a brief description of all guidelines used in the conduction of this study. [file spectrum.00291-25-s0001.docx]

***Supplementary Materials***

Table S1 Susceptibility Report

|  |  | **n=121** | **%** |
| --- | --- | --- | --- |
| **Amikacin** | |  |  |
|  | Sensitive | 12 | 9.9 |
|  | Resistant | 42 | 34.7 |
|  | Intermediate | 3 | 2.5 |
|  | Not reported | 64 | 52.9 |
| **Trimethoprim/Sulfamethoxazole (Bactrim/Septrin)** | | | |
|  | Sensitive | 16 | 13.2 |
|  | Resistant | 78 | 64.5 |
|  | Not reported | 27 | 22.3 |
| **Piperacillin/Tazobactam (Tazocin)** | | | |
|  | Sensitive | 7 | 5.8 |
|  | Resistant | 90 | 74.4 |
|  | Intermediate | 1 | .8 |
|  | Not reported | 23 | 19.0 |
| **Nitrofurantoin** | |  |  |
|  | Sensitive | 5 | 4.1 |
|  | Resistant | 44 | 36.4 |
|  | Not reported | 72 | 59.5 |
| **Meropenem** | |  |  |
|  | Sensitive | 15 | 12.4 |
|  | Resistant | 65 | 53.7 |
|  | Intermediate | 2 | 1.7 |
|  | Not reported | 39 | 32.2 |
| **Imipenem** | |  |  |
|  | Sensitive | 27 | 22.3 |
|  | Resistant | 52 | 43.0 |
|  | Intermediate | 15 | 12.4 |
|  | Not reported | 27 | 22.3 |
| **Gentamicin** | |  |  |
|  | Sensitive | 51 | 42.1 |
|  | Resistant | 58 | 47.9 |
|  | Not reported | 12 | 9.9 |
| **Ciprofloxacin** | |  |  |
|  | Sensitive | 3 | 2.5 |
|  | Resistant | 114 | 94.2 |
|  | Intermediate | 1 | .8 |
|  | Not reported | 3 | 2.5 |
| **Ceftriaxone** | |  |  |
|  | Sensitive | 14 | 11.6 |
|  | Resistant | 72 | 59.5 |
|  | Not reported | 35 | 28.9 |
| **Ceftazidime** | |  |  |
|  | Sensitive | 3 | 2.5 |
|  | Resistant | 92 | 76.0 |
|  | Not reported | 26 | 21.5 |
| **Ceftazidime/Avibactam (Avycaz)** | | | |
|  | Sensitive | 16 | 13.2 |
|  | Resistant | 7 | 5.8 |
|  | Not reported | 98 | 81.0 |
| **Cefoxitin** | |  |  |
|  | Resistant | 66 | 54.5 |
|  | Not reported | 55 | 45.5 |
| **Cefepime** | |  |  |
|  | Sensitive | 3 | 2.5 |
|  | Resistant | 90 | 74.4 |
|  | Not reported | 28 | 23.1 |
| **Ampicillin** | |  |  |
|  | Resistant | 91 | 75.2 |
|  | Not reported | 30 | 24.8 |
| **Amoxicillin/ Clavulanic acid** | | | |
|  | Sensitive | 8 | 6.6 |
|  | Resistant | 74 | 61.2 |
|  | Not reported | 39 | 32.2 |
| **Ertapenem** | |  |  |
|  | Sensitive | 6 | 5.0 |
|  | Resistant | 29 | 24.0 |
|  | Not reported | 86 | 71.1 |
| **Tetracycline** | |  |  |
|  | Sensitive | 1 | .8 |
|  | Resistant | 5 | 4.1 |
|  | Intermediate | 1 | .8 |
|  | Not reported | 114 | 94.2 |
| **Tigecycline** | |  |  |
|  | Sensitive | 29 | 24.0 |
|  | Resistant | 16 | 13.2 |
|  | Intermediate | 25 | 20.7 |
|  | Not reported | 51 | 42.1 |
| **Cefalotin** | |  |  |
|  | Resistant | 63 | 52.1 |
|  | Not reported | 58 | 47.9 |
| **Vancomycin** | |  |  |
|  | Sensitive | 3 | 2.5 |
|  | Resistant | 8 | 6.6 |
|  | Not reported | 110 | 90.9 |
| **Ceftolozane/Tazobactam (Zerbaxa)** | | | |
|  | Sensitive | 2 | 1.7 |
|  | Resistant | 3 | 2.5 |
|  | Not reported | 116 | 95.9 |
| **Levofloxacin** | |  |  |
|  | Resistant | 24 | 19.8 |
|  | Not reported | 97 | 80.2 |
| **Tobramycin** | |  |  |
|  | Resistant | 13 | 10.7 |
|  | Not reported | 108 | 89.3 |
| **Linezolid** | |  |  |
|  | Sensitive | 11 | 9.1 |
|  | Not reported | 110 | 90.9 |
| **Colistin** | |  |  |
|  | Sensitive | 3 | 2.5 |
|  | Resistant | 2 | 1.7 |
|  | Not reported | 116 | 95.9 |
| **Oxacillin** | |  |  |
|  | Resistant | 1 | .8 |
|  | Not reported | 120 | 99.2 |
| **Ampicillin/sulbactam** | | |  |
|  | Resistant | 12 | 9.9 |
|  | Not reported | 109 | 90.1 |
| **Extended spectrum beta-lactamase (ESBL) (n=36)** | | | |
|  | positive | 22 | 61.1 |
|  | negative | 14 | 38.9 |
| **Minocycline** | |  |  |
|  | Resistant | 3 | 2.5 |
|  | Not reported | 118 | 97.5 |
| **Gene** |  |  |  |
|  | OXA 48 | 13 | 43.3 |
|  | NDM | 2 | 6.7 |
|  | KPC | 3 | 10.0 |
|  | KPC / NDM | 4 | 13.3 |
|  | OXA 48 / NDM | 8 | 26.7 |

Table S2 The Relation Between the Guidelines’ Recommended Antibiotics and the Susceptibility Report

|  | **The pathogen is susceptible to the recommended antibiotics** | **The pathogen is resistant to the recommended antibiotics** | **The recommended antibiotics are not reported in the susceptibility report** |
| --- | --- | --- | --- |
| **Vancomycin (n=47)** | | | |
| n | 2 | 1 | 44 |
| % | 4.3 | 2.1 | 93.6 |
| **Doxycycline (n=1)** | | | |
| n | 0 | 0 | 1 |
| % | 0 | 0 | 100.0 |
| **Meropenem (n=66)** | | | |
| n | 16 | 40 | 10 |
| % | 24.2 | 60.6 | 15.2 |
| **Piperacillin/Tazobactam (Tazocin) (n=51)** | | | |
| n | 5 | 41 | 5 |
| % | 9.8 | 80.4 | 9.8 |
| **Trimethoprim/Sulfamethoxazole (Bactrim/Septrin) (n=1)** | | | |
| n | 0 | 0 | 1 |
| % | 0 | 0 | 100.0 |
| **Colistin (n=11)** | | | |
| n | 0 | 0 | 11 |
| % | 0 | 0 | 100.0 |
| **Cefepime (n=1)** | | | |
| n | 0 | 1 | 0 |
| % | 0 | 100.0 | 0 |
| **Gentamicin (n=3)** | | | |
| n | 1 | 0 | 2 |
| % | 33.3 | 0 | 66.7 |
| **Ceftriaxone (n=7)** | | | |
| n | 2 | 3 | 2 |
| % | 28.6 | 42.9 | 28.6 |
| **Aztreonam (Azactam) (n=1)** | | | |
| n | 0 | 0 | 1 |
| % | 0 | 0 | 100.0 |
| **Azithromycin (n=3)** | | | |
| n | 0 | 0 | 3 |
| % | 0 | 0 | 100.0 |
| **Silver Sulfadiazine (n=1)** | | | |
| n | 1 | 0 | 0 |
| % | 100.0 | 0 | 0 |
| **Amikacin (n=15)** | | | |
| n | 3 | 4 | 8 |
| % | 20.0 | 26.7 | 53.3 |

Table S3 The Relation Between the Administered Empirical Treatment and the Susceptibility Report

|  |  | n | % |
| --- | --- | --- | --- |
| **Vancomycin (n=38)** | | | |
|  | The pathogen was susceptible to the ADMINISTERED antibiotic | 1 | 2.1 |
|  | The pathogen was resistant to the ADMINISTERED antibiotic | 3 | 6.3 |
|  | The ADMINISTERED antibiotic was not reported | 44 | 91.7 |
| **Doxycycline (n=3)** | | | |
|  | The pathogen was susceptible | 0 | 0 |
|  | The pathogen was resistant | 0 | 0 |
|  | The ADMINISTERED antibiotic was not reported | 3 | 100.0 |
| **Meropenem (n=68)** | | | |
|  | The pathogen was susceptible | 18 | 26.5 |
|  | The pathogen was resistant | 41 | 60.3 |
|  | The ADMINISTERED antibiotic was not reported | 9 | 13.2 |
| **Piperacillin/Tazobactam (Tazocin) (n=30)** | | | |
|  | The pathogen was susceptible | 5 | 16.7 |
|  | The pathogen was resistant | 22 | 73.3 |
|  | The ADMINISTERED antibiotic was not reported | 3 | 10.0 |
| **Ceftazidime (n=1)** | | | |
|  | The pathogen was susceptible | 0 | 0 |
|  | The pathogen was resistant | 1 | 100.0 |
|  | The ADMINISTERED antibiotics was not reported | 0 | 0 |
| **Avycaz (n=18)** | | | |
|  | The pathogen was susceptible | 15 | 83.3 |
|  | The pathogen was resistant | 0 | 0 |
|  | The ADMINISTERED antibiotic was not reported | 3 | 16.7 |
| **Trimethoprim/Sulfamethoxazole (Bactrim/Septrin) (n=2)** | | | |
|  | The pathogen was susceptible | 0 | 0 |
|  | The pathogen was resistant | 1 | 50.0 |
|  | The ADMINISTERED antibiotic was not reported | 1 | 50.0 |
| **Colistin (n=26)** | | | |
|  | The pathogen was susceptible | 2 | 7.7 |
|  | The pathogen was resistant | 0 | 0 |
|  | The ADMINISTERED antibiotic was not reported | 24 | 92.3 |
| **Cefepime (n=1)** | | | |
|  | The pathogen was susceptible | 0 | 0 |
|  | The pathogen was resistant | 1 | 100.0 |
|  | The ADMINISTERED antibiotic was not reported | 0 | 0 |
| **Ciprofloxacin (n=5)** | | | |
|  | The pathogen was susceptible | 1 | 20.0 |
|  | The pathogen was resistant | 4 | 80.0 |
|  | The ADMINISTERED antibiotic was not reported | 0 | 0 |
| **Tigecycline (n=6)** | | | |
|  | The pathogen was susceptible | 1 | 16.7 |
|  | The pathogen was resistant | 1 | 16.7 |
|  | The ADMINISTERED antibiotic was not reported | 4 | 66.7 |
| **Gentamicin (n=3)** | | | |
|  | The pathogen was susceptible | 3 | 100.0 |
|  | The pathogen was resistant | 0 | 0 |
|  | The ADMINISTERED antibiotic was not reported | 0 | 0 |
| **Ceftriaxone (n=8)** | | | |
|  | The pathogen was susceptible | 2 | 25.0 |
|  | The pathogen was resistant | 4 | 50.0 |
|  | The ADMINISTERED antibiotic was not reported | 2 | 25.0 |
| **Aztreonam (Azactam) (n=7)** | | | |
|  | The pathogen was susceptible | 7 | 100.0 |
|  | The pathogen was resistant | 0 | 0 |
|  | The ADMINISTERED antibiotic was not reported | 0 | 0 |
| **Metronidazole (n=1)** | | | |
|  | The pathogen was susceptible | 0 | 0 |
|  | The pathogen was resistant | 0 | 0 |
|  | The ADMINISTERED antibiotic was not reported | 1 | 100.0 |
| **Azithromycin (n=3)** | | | |
|  | The pathogen was susceptible | 0 | 0 |
|  | The pathogen was resistant | 0 | 0 |
|  | The ADMINISTERED antibiotic was not reported | 3 | 100.0 |
| **Daptomycin (n=2)** | | | |
|  | The pathogen was susceptible | 0 | 0 |
|  | The pathogen was resistant | 0 | 0 |
|  | The ADMINISTERED antibiotic was not reported | 2 | 100.0 |
| **Silver sulfadiazine (n=1)** | | | |
|  | The pathogen was susceptible | 1 | 100.0 |
|  | The pathogen was resistant | 0 | 0 |
|  | The ADMINISTERED antibiotic was not reported | 0 | 0 |
| **Linezolid (n=2)** | | | |
|  | The pathogen was susceptible | 1 | 50.0 |
|  | The pathogen was resistant | 0 | 0 |
|  | The ADMINISTERED antibiotic was not reported | 1 | 50.0 |
| **Amikacin (n=7)** | | | |
|  | The pathogen was susceptible | 3 | 42.9 |
|  | The pathogen was resistant | 0 | 0 |
|  | The ADMINISTERED antibiotic was not reported | 4 | 57.1 |
| **Ertapenem (n=1)** | | | |
|  | The pathogen was susceptible | 0 | 0 |
|  | The pathogen was resistant | 0 | 0 |
|  | The ADMINISTERED antibiotic was not reported | 1 | 100.0 |

List of the Ministry of National Guard Health Affairs (MNGHA) antimicrobial guidelines used for concordance evaluation

1. Antimicrobial Guidelines

- Purpose:
  - To define each disease with the most common symptoms and a list of the most common causative pathogens, along with listed options for empirical therapy
- Key Sections:
  - Abdominal Infections
  - Brucellosis
  - Central Nervous System Infections
  - ﻿Device-Associated Bloodstream Infections
  - Upper Respiratory tract Infections
  - Pulmonary Infections
  - Sexually Transmitted Diseases (STDs)
  - Skin and Soft Tissue Infections
  - Tuberculosis
  - Urinary Tract Infections
  - Febrile Neutropenia
  - Infective Endocarditis
  - ﻿Antimicrobial Surgical Prophylaxis

1. ﻿Adult Renal Dose Adjustment For Most Commonly Used Antimicrobials

- Purpose: ﻿
  - To regulate, standardize, and provide guidelines for dosing of most commonly used antimicrobials based on renal function
  - To enhance patient care through optimization of antimicrobial dosing
  - To reduce adverse events associated with inappropriate antimicrobial use
- Key Sections:
  - Tables with dosing regimens adjusted for renal function

1. Approved Indications for Restricted Antimicrobials

- Purpose:
  - ﻿To ensure optimal use of the restricted antimicrobials through clearly identifying MNGHA approved indications of use and situations where these antimicrobials should not be used or approved
- Key Sections:
  - Definitions of restriction levels
  - Prescribing authority structure
  - Criteria for approval

1. Aminoglycoside Dosing and Monitoring Guidelines

- Purpose:
  - ﻿To regulate, standardize, and provide guidelines for aminoglycoside administration
  - To enhance patient care through optimization of aminoglycoside dosing
  - To reduce adverse events associated with aminoglycoside administration
- Key Sections:
  - Background and rationale for aminoglycosides usage
  - Tables with dosing regimens adjusted for renal function

1. ﻿Beta Lactam Administration via Prolonged Infusion Guidelines

- Purpose:
  - To regulate, standardize, and provide guidelines for beta-lactam administration via prolonged infusion for patients
  - To enhance patient care through optimization of drug concentration target attainment at infection site
- Key Sections:
  - Background and rationale for using beta-lactam antimicrobials
  - Tables with dosing regimens adjusted for renal function

1. Colistin Dosing Guidelines

- Purpose:
  - ﻿To regulate, standardize, and provide guidelines for colistin administration
  - To enhance patient care through optimization of colistin dosing
  - To reduce adverse events associated with colistin administration
- Key Sections:
  - Background and rationale for the use of colistin
  - Table with dosing regimens adjusted for renal function

1. Pharmacological Management of Metalobetalactamase Producing Enterobacterales

- Purpose:
  - ﻿To ensure optimal use of the combination of ceftazidime/avibactam with aztreonam for the treatment of organisms that produce metalobetalactamases
- Key Sections:
  - Background and rationale for the use of of the combination of ceftazidime/ avibactam with aztreonam
  - Table with dosing regimens adjusted for renal function

1. Parenteral Vancomycin Guidelines

- Purpose:
  - ﻿To regulate, standardize, and provide guidelines for vancomycin administration
  - To enhance patient care through optimization of vancomycin dosing and monitoring
  - To reduce adverse events associated with vancomycin use
- Key Sections:
  - Background and rationale for vancomycin administration
  - Tables with dosing regimens adjusted for renal function
